# Inferring the temperature dependence of population parameters: the effects of experimental design and inference algorithm

**DOI:** 10.1002/ece3.1309

**Published:** 2014-12-02

**Authors:** Gian Marco Palamara, Dylan Z Childs, Christopher F Clements, Owen L Petchey, Marco Plebani, Matthew J Smith

**Affiliations:** 1 Department of Evolutionary Biology and Environmental Studies, University of ZurichWintherthurerstrase 190, CH-8057, Zurich, Switzerland; 2 Department of Animal and Plant Sciences, University of SheffieldSheffield, S10 2TN, UK; 3 Computational Science Laboratory, Microsoft ResearchCambridge, CB1 2FB, UK

**Keywords:** Activation energy, Arrhenius equation, maximum likelihood, MCMC, metabolic theory, microcosm experiments, state space models, stochastic simulations

## Abstract

Understanding and quantifying the temperature dependence of population parameters, such as intrinsic growth rate and carrying capacity, is critical for predicting the ecological responses to environmental change. Many studies provide empirical estimates of such temperature dependencies, but a thorough investigation of the methods used to infer them has not been performed yet. We created artificial population time series using a stochastic logistic model parameterized with the Arrhenius equation, so that activation energy drives the temperature dependence of population parameters. We simulated different experimental designs and used different inference methods, varying the likelihood functions and other aspects of the parameter estimation methods. Finally, we applied the best performing inference methods to real data for the species *Paramecium caudatum*. The relative error of the estimates of activation energy varied between 5% and 30%. The fraction of habitat sampled played the most important role in determining the relative error; sampling at least 1% of the habitat kept it below 50%. We found that methods that simultaneously use all time series data (direct methods) and methods that estimate population parameters separately for each temperature (indirect methods) are complementary. Indirect methods provide a clearer insight into the shape of the functional form describing the temperature dependence of population parameters; direct methods enable a more accurate estimation of the parameters of such functional forms. Using both methods, we found that growth rate and carrying capacity of *Paramecium caudatum* scale with temperature according to different activation energies. Our study shows how careful choice of experimental design and inference methods can increase the accuracy of the inferred relationships between temperature and population parameters. The comparison of estimation methods provided here can increase the accuracy of model predictions, with important implications in understanding and predicting the effects of temperature on the dynamics of populations.

## Introduction

Explaining the distribution and abundance of organisms requires knowledge of the environmental dependence of organismal properties (Hall et al. 1992; Ives 1995), including biological rates such as birth and death rate (Volkov et al. 2003). Furthermore, predicting the effects of environmental change on populations benefits from understanding the environmental dependence of biological processes (Ives 1995; Thomas et al. 2004; Deutsch et al. 2008; Vasseur et al. 2014). Empirical relationships between the rates of physiological processes and one particularly important environmental variable, temperature, have been documented for many processes and taxa (Gillooly et al. 2001, 2002; Dell et al. 2010), including rates of food ingestion by individuals (Englund et al. 2011; O'Connor et al. 2011; Dell et al. 2014), rates of population growth (Savage et al. 2004), and rates of various ecosystem processes (Ernest et al. 2003; Allen et al. 2005; Yvon-Durocher et al. 2012). These and other relationships have been used to predict effects of temperature on population dynamics (Vasseur and McCann 2005). The overall aim of this paper is to provide improved inference methods for estimating such relationships.

Methods used to infer the population parameters from time series data typically range from classic maximum likelihood estimation (Hilborn 1997) to Bayesian inference for partially observed Markov processes (Knape and De Valpine 2012; Dennis and Ponciano 2014). When estimating population parameters, one needs a description of the sampling error associated with any experiment or field survey, as well as an explicit model of the dynamics (De Valpine and Hastings 2002; Dennis et al. 2006; Dennis and Ponciano 2014). An important decision is thus whether inference method should explicitly account for the sampling process, that is, the process that provides the actual counts of number of individuals. Unless the entire habitat is sampled (so that every individual is counted), the observed number of individuals will be a sample of the actual abundance (De Valpine and Hastings 2002; Dennis et al. 2006; Ross 2012) and not including sampling error can lead to erroneous parameter estimates (Ionides et al. 2006). Fitting stochastic population dynamic models to observed data while taking into account sampling error is a nontrivial endeavor (Ionides et al. 2006; Ross 2012). Hence, it would be very useful to know when such an approach is necessary and when a simpler approach (e.g., a deterministic model with no accounting for sampling error) provides sufficiently accurate and precise estimates.

We focus on improving inference of the relationship between two population parameters (intrinsic growth rate *r* and carrying capacity *K*) and temperature. The Arrhenius law, which was originally proposed to describe the temperature dependence of the specific reaction rate constant in chemical reactions (Van't Hoff 1884; Arrhenius 1889), is used to describe the temperature dependence of whole-organism metabolic rates such as growth rate (Schoolfield et al. 1981). The Arrhenius law predicts that the natural logarithm of mass-corrected metabolic rates is a linear function of the inverse absolute temperature. The slope of this relationship gives the activation energy of metabolism (Arrhenius 1889; Schoolfield et al. 1981), and the intercept gives the natural logarithm of the normalization constant (Brown et al. 2004). The temperature dependence of *r* has been studied extensively (Dell et al. 2010; Corkrey et al. 2012), especially in microbes (Monod 1942; Weisse and Montagnes 1998; Weisse et al. 2002; Jang and Morin 2004; Price and Sowers 2004; Krenek et al. 2011, 2012), rotifers (Montagnes et al. 2001), algae (Montagnes and Franklin 2001), and insects (Irlich et al. 2009; Amarasekare and Sifuentes 2012). The temperature dependence of *K* has received less attention (Yodzis and Innes 1992; Brown et al. 2004; Savage et al. 2004; Vasseur and McCann 2005). In this study, we focus on the statistical methods used to infer such temperature rate relationships. We do not enter the debate about the validity of Arrhenius law (Knies and Kingsolver 2010) or on the exact value of activation energy (Glazier 2006), although in the discussion we will indicate how our insights can be used to address these debates.

Data needed to assess the temperature dependence of population parameters come in the form of time series collected at different (fixed) temperatures (Jang and Morin 2004; Beveridge et al. 2010; Krenek et al. 2012; Leary et al. 2012). This is performed in experiments in which single-species populations are grown at a variety of temperatures, starting from very low abundances, until carrying capacity is reached. Population size is recorded with a certain temporal frequency, most often from a subsample of the total habitat (i.e., the population is sampled), thus providing a time series for each temperature. The estimates of *r* and *K* obtained at each temperature over a range of temperatures are used to estimate activation energy through the Arrhenius law (Gillooly et al. 2002; Savage et al. 2004). Although our study assumes a temperature range for which the Arrhenius law is appropriate, the results will generalize to a wider range of temperatures. We term the use of this approach an “indirect method” of estimating the activation energy. This is, to date, the most common approach to estimating activation energy from growth processes (Weisse et al. 2002; Price and Sowers 2004; Savage et al. 2004; Angiletta 2006; Huang et al. 2011; Krenek et al. 2011, 2012; Corkrey et al. 2012) and from other processes (Rall et al. 2009; Englund et al. 2011). An alternative approach, which we term the “direct method”, is to directly fit a model of the temperature dependence of population dynamics to the entire dataset, that is, to fit to population dynamics from all the temperature treatments simultaneously. Based on limited previous comparisons of indirect and direct estimation methods, we expect the direct method to have higher accuracy and precision than the indirect method (Schoolfield et al. 1981; Price and Sowers 2004), because it is combining more information directly in the inference process to infer fewer parameters. As well as making this comparison, we illustrate the ecological consequences of the observed differences in accuracy and precision.

**Figure 1:**
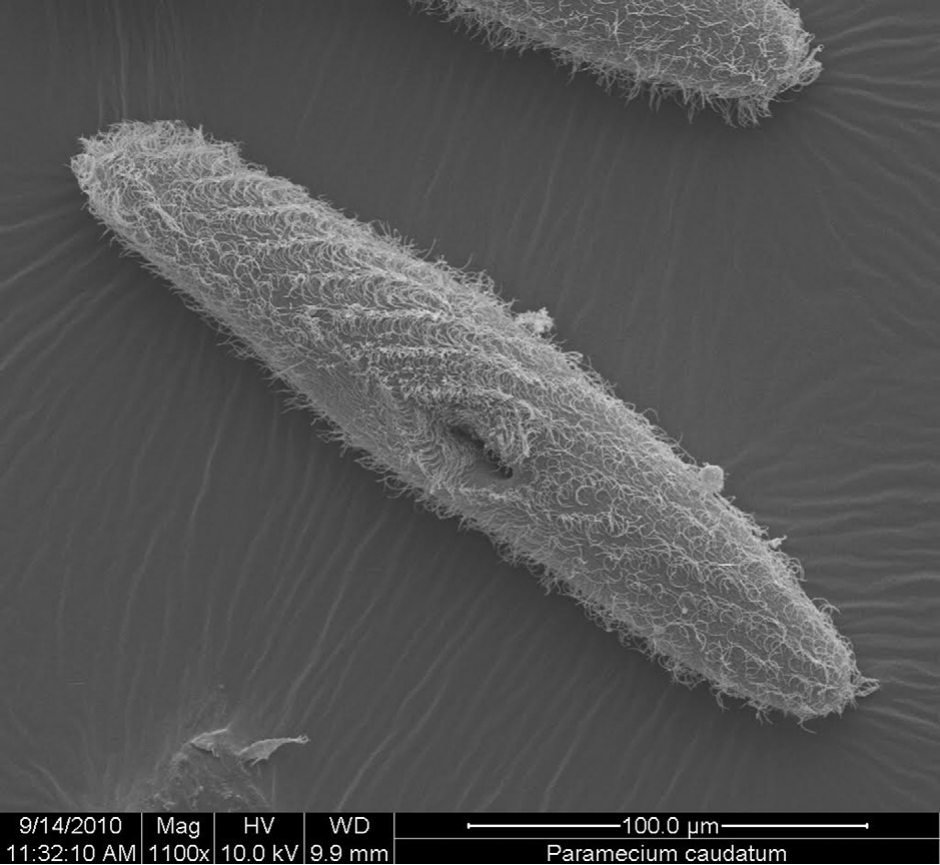
Picture of the living freshwater species *Paramecium caudatum* (courtesy of Dr. Renate Radek).

In addition to choices about inference methods, a researcher makes choices about the design of the experiments used to produce the observed data. Here, we assess the importance of different experimental designs and inference methods on the ability to infer activation energy from time series data on single-species experimental microcosms. We assess the performance of different inference methods given particular choices of experimental designs by estimating the activation energy of simulated population data. We also demonstrate application of the methods to real data from experiments with *Paramecium caudatum*, a well-studied freshwater protist species (Krenek et al. 2011, 2012; Fig. 1). We used only one species as a case study because the focus of our study is methodological, rather than descriptive. We chose *Paramecium caudatum* because it shows population growth that is well captured by the stochastic logistic equation (Leary and Petchey 2009). We provide advice for experimentalists about the most relevant factors affecting the precision and the accuracy of the estimates of activation energy for different inference methods.

To our knowledge, there has been no thorough and systematic exploration of the relative importance of these issues (i.e., influence of experimental design, sampling design, model type, and inference method) for the accuracy and precision of estimates of environmental dependence of ecological parameters such as the temperature dependence of intrinsic growth rate and carrying capacity. The methods are illustrated with estimation of *r* and *K*, but can be generalized to estimation of the activation energy of other biological rates, such as maximum consumption rate (Rall et al. 2009; Englund et al. 2011), and effects of environmental variables other than temperature, for example, nutrient availability (Weisse et al. 2002; Price and Sowers 2004).

## Methods

We describe population dynamics using a continuous time, stochastic logistic model (Nåsell 2001), a generalization of the deterministic logistic equation in continuous time (McKane and Newman 2004; Gardinier 2009). Stochastic models can provide fundamentally different results from their deterministic counterparts (Ebenman et al. 2004; McKane and Newman 2005) and provide a more detailed description of the mechanisms affecting population dynamics (Black and McKane 2012). For example, the carrying capacity (*K*) in the deterministic logistic equation is the equilibrium population density of a given species, namely the maximum sustainable population size given the available resources (Malthus 1798; Turchin 2003). Conversely in stochastic logistic growth models, *K* represents the mean of a long-term stationary distribution around which the population fluctuates (Nåsell 2001; Dennis et al. 2006).

We performed a simulation study to assess the importance of experimental protocols and inference methods on the ability to estimate the activation energy for the temperature dependence of population parameters. This involved simulating population dynamic data using a model with known activation energy in section “Model and simulations”, and comparison of this true activation energy to that obtained by various inference methods in section “Parameter inference”. We illustrated the best performing methods by estimating activation energy from real population dynamic data of a free-living freshwater protist species, *Paramecium caudatum* in section “Case study”.

### Model and Simulations

We used a simple stochastic birth and death processes (BDP) model to generate time series data of population dynamics


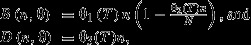
(1)

where 0 ≤ *n* ≤ *N* is the (integer) number of individuals, *N* is population size at which there is zero probability of births, θ_1_ and θ_3_ are the per capita birth and death rates in the absence of density dependence, respectively (units: day^−1^), θ_2_ controls the strength of density-dependent effects on the probability of births (dimensionless), and (*T*) indicates that all θ parameters are dependent on temperature, *T* (measured in Kelvin). We used the BDP 1 because it allows to take into account all biological mechanisms affecting population dynamics (for more details on the model see section “Details on the formulation of the stochastic model” in Supporting information); for simplicity, we assume that density dependence only affects probabilities of births, although in reality, density dependence likely influences the probability of both births and deaths (i.e., both births and deaths in process 1 would be influenced by *N*). We introduce temperature dependence to the θ parameters using the Arrhenius equation (Gillooly et al. 2001)


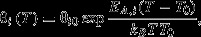
(2)

where *i* = 1,2,3 denotes the population parameter in the BDP 1, *E*_*A,i*_ is the activation energy (units: ElectronVolts = eV) for parameter θ_*i*_, *k*_*B*_ is the Boltzmann constant, and *T*_0_ is a reference baseline temperature, which we assume to be 301.5 K (28°C). For most of our analyses, we assume the same *E*_*A,i*_ for all parameters.

The mean population abundance over time follows the logistic equation


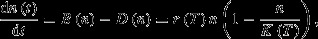
(3)

where *r*(*T*) = θ_1_(*T*) – θ_3_(*T*) is the maximum population growth rate and 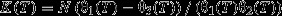 is the carrying capacity (Nåsell 2001). The temperature dependencies of growth rate and carrying capacity are thus


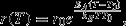
(4)


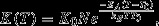
(5)

where *r*_0_ = θ_01_ – θ_30_ and 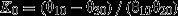 are the growth rate and carrying capacity at *T*_0_. Expressions 4 and 5 indicate that growth rate and carrying capacity should increase and decrease with temperature, respectively (Savage et al. 2004).

**Figure 2:**
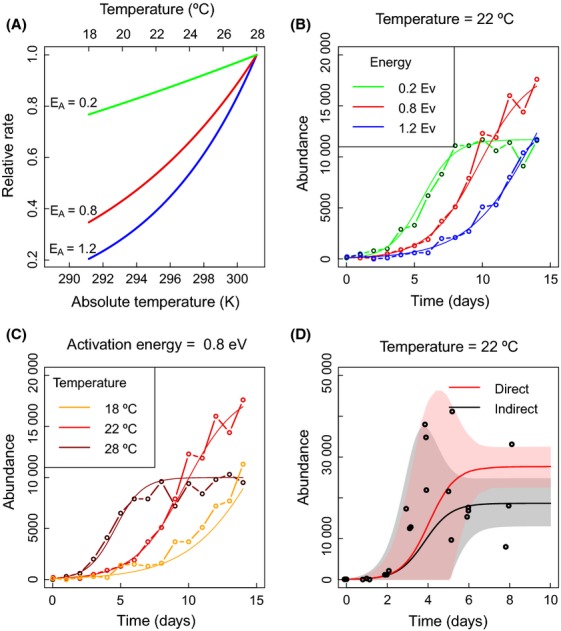
Example of temperature dependence of a rate for three different activation energies (Panel A) standardized to have the same value at 301.15 K (Huey and Kingsolver 2011). Panels B and C show the effect of activation energy (panel B) and temperature (panel C) on time series originated by the BDP 1 with parameters scaled using equation 2. The simulated time series all have an initial condition of 100 individuals are sampled everyday for 15 days (TIMESAMP = 15) and are subjected to demographic noise and sampling error (FRACSAMP = 0.01). The continuous lines show the deterministic solution 13. Panel D shows real time series data (black dots) for 3 replicates of *Paramecium caudatum* monocultures (maximum FRACSAMP = 0.001). We show the corresponding fitted means (continuous lines) and modeled variances (shaded areas) using both direct (red) and indirect (black) methods. The estimated activation energies are shown in Figure 6.

We simulated the process 1 and the relations 2 using the well-known Gillespie algorithm (Gillespie 1976) (see Fig. 2 for examples). This produced continuous time series recording the exact times of individual birth and death events. To make simulated data more representative of experimental data, we then sampled population size at discrete times as if only a fraction of the population had been sampled and counted (examples are shown in Fig. 2). To simulate sampling, we assumed that the numbers measured were drawn from a Poisson distribution centered on the expected number of individuals contained in a sample from the population, where the sample size FRACSAMP is the fraction of the habitat searched. We do not include an additional source of error from the imperfect ability of observers to count all individuals in a sample; thus, demographic stochasticity and sampling error associated with the fraction of the habitat searched (FRACSAMP) are the only two sources of stochasticity in our simulated experimental data.

We chose parameter values for equations 1 and 2 that lead to similar simulated population dynamics to those observed in laboratory experiments (see Fig. 2) and that are consistent with previously published values (Savage et al. 2004). We set the reference temperature *T*_0_ = 28°C and scaled the other population parameters relative to their probabilities at that temperature: θ_1_(*T*_0_) = 1.5 day^−1^, 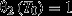, θ_3_(*T*_0_) = 0.5 day^−1^. The population size at which the probability of births is zero, *N*, was fixed throughout this study to *N* = 15,000 individuals. The importance of this value is detailed in the discussion and here was chosen in order to represent a typical laboratory experiment with a microcosm of 10 mL.

These choices lead to a maximum population growth rate of *r*(*T*_0_) = 1 day^−1^ and a minimum carrying capacity of *K*(*T*_0_) = 10,000 individuals. All simulations began with an initial population size of *n*_0_ = 100 individuals and lasted 15 days. We simulated equations 1 and 2 under 81 different sets of experimental conditions, representing the range of experimental strategies likely to be considered when conducting laboratory experiments to estimate activation energy. These 81 experiments arise from a fully factorial experimental design in which four factors are varied, with three different values each. We varied

- The number of different temperatures considered, TEMPSAMP. We generated time series at 11 different temperatures from 18 to 28°C in steps of 1°C but varied the numbers of different temperatures used in the estimation of activation energy: either using all 11 temperatures, using only six different temperatures (from 18 to 28°C in steps of 2°C), or using just three different temperatures (18, 23 and 28°C). Those temperature gradients were chosen in order to capture the temperature range where we expect the Arrhenius law 2 to be valid. Note that if a wider range of temperatures were to be investigated, then the rates may start to decrease at higher temperatures, requiring fitting of a hump-shaped function rather than the Arrhenius equation (Corkrey et al. 2012; Krenek et al. 2012).
- The number of replicate experiments at each temperature and activation energy, REPS. We considered one, three, or five replicates at each temperature. While estimation using one replicate per temperature is possible, from three to five are typically used in experiments where population time series are recorded (Leary and Petchey 2009; Krenek et al. 2011).
- The number of samples taken during an experiment, TIMESAMP. We considered once every three days (TIMESAMP = 5), twice every three days (TIMESAMP = 10), or once a day (TIMESAMP = 15) over the course of each 15 days experiment. Fifteen days were sufficient to capture both the growth phase and the equilibrium phase (carrying capacity) of the population dynamics.
- The fraction of habitat sampled, FRACSAMP. We considered 1%, 0.5%, and 0.1% of the entire habitat (FRACSAMP = 0.01, 0.005 and 0.001), reproducing the typical search effort of experiments (De Valpine and Hastings 2002; Dennis et al. 2006).

For each experimental design, we then estimate activation energy using different methods.

### Parameter inference

To conduct parameter inference, we need a mathematical function defining the probability of a set of parameters given the data, that is, the likelihood function. We compared different methods for inferring activation energy (summarized in Table 1) using five different likelihood functions (for details on the derivation of the likelihood functions see section “Likelihoods and inference” in Supporting information). The model underpinning methods M1 and M2 is the solution of equation 3, that is, the likelihood function is parameterized using only the mean population abundance overtime, assuming that the dynamics are deterministic. The second model (underpinning methods M3–M6) assumes that the dynamics are demographically stochastic but that there is no sampling error; the correspondent likelihood function is parameterized using both the mean and the variance of population abundance (see section “Details on the formulation of the stochastic model” in Supporting information and Ross et al. 2009 for the diffusion approximation used in the derivation of the population variance). In methods M7–M8, we add to the likelihood function of methods M3–M6 a correction taking into account for the sampling error.

**Table 1:** Methods to infer activation energy

Inference method	Likelihood function	Parameter used	Estimate	Method	Corr.	Comp. time (h)
M1	*L*_*phen*_(**Θ**) (15)	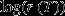	INDIRECT	MLE	NO	0.5*
M2	*L*_*phen*_(**Θ**) (15)	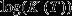	INDIRECT	MLE	NO	0.5*
M3	*L*_1_(θ^′^) (16)	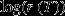	INDIRECT	MLE	NO	0.5*
M4	*L*_1_(θ^′^) (16)	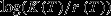	INDIRECT	MLE	NO	0.5*
M5	*L*_1_(θ^′^) (16)	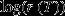	INDIRECT	MCMC	NO	1
M6	*L*_1_(θ^′^) (16)	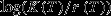	INDIRECT	MCMC	NO	1
M7	*L*_2_(θ^′^) (18)	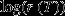	INDIRECT	MCMC	YES	2
M8	*L*_2_(θ^′^) (18)	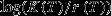	INDIRECT	MCMC	YES	2
M9	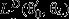 (19)	log(*E*_*A*_)	DIRECT	MCMC	NO	1.5
M10	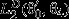 (20)	log(*E*_*A*_)	DIRECT	MCMC	YES	2.5

Column three (parameter used) specifies which parameter is used to obtain the estimate of activation energy. Column five (Method) refers to the statistical framework used, that is, MLE (maximum likelihood estimation) or MCMC (Markov chain Monte Carlo). Column six (Corr.) states whether the correction for sampling error was implemented (YES) or not (NO). The last column of the table shows computational times of each method when inferring activation energy using the same simulated data for all inference methods (FRACSAMP = 0.01, REP = 5, TIMESAMP = 10, TEMPSAMP = 11) for a fixed activation energy (*E*_*A*_ = 0.2 eV). The computational time was measured on a desktop computer whose processor is Intel(R) Xenon(R) E5645 2.4 GHz, with installed RAM of 12 GB. The numbers denoted by * are widely variable even on the same operating system. In fact, frequently, the algorithm returns NA for the mean and or the variance of the parameter estimates and the time taken to obtain the parameter estimates are highly variable. The numbers reported are chosen as representative from the runs that reported real numbers for the mean and variance of the parameter estimates.

Methods M1–M8 are defined as indirect as they adopt the common approach of inferring activation energy indirectly, that is, population growth rates (*r*) or carrying capacities (*K*) are inferred at different temperatures. Activation energy is then deduced from the relationship between these parameters and the inverse energy 1/*k*_*B*_*T* (see Fig. 2B) given by


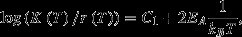
(6)


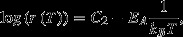
(7)

where *C*_1_ = log(*N*/θ_10_/θ_20_) − 2*E*_*A*_*k*_*B*_/*T*_0_ and 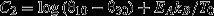 are two temperature-independent constants. Activation energy is the slope of these relationships, derived using standard linear regression between the logarithm of the parameters of the logistic equation and the inverse temperature (Schoolfield et al. 1981; see Fig. 2B), as it has been extensively performed in previous studies (Schoolfield et al. 1981; Gillooly et al. 2001, 2002; Savage et al. 2004).

The other approach we take is to infer activation energy directly. Method M9 is a generalization of methods M5–M6, and its likelihood is obtained by summing the likelihood underpinning methods M5–M6 over all observed temperatures. Similarly, method M10 is a generalization of methods M7–M8 and takes into account the sampling error. The likelihood of method M10 is obtained by summing the likelihoods of models M7–M8 over all observed temperatures (see section “Likelihoods and inference” for more details on the direct methods). The indirect methods used to infer activation energy are characterized by the choice of one parameter (growth rate or carrying capacity) whose temperature dependence (relations 6 and 7) provides an estimate of activation energy. Direct methods, on the other hand, provide an estimate of activation energy from the global temperature dependency of all the parameters of model 1.

For each inference algorithm and experiment, we measured the relative error (*R*) and precision (*P*) of the estimate given by


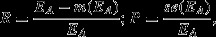
(8)

where *E*_*A*_ is the real value of activation energy used to produce the simulated data, *m*(*E*_*A*_) is the mean of the estimate, and *se*(*E*_*A*_) is the standard error of the estimate. The accuracy of the estimates of activation energy is given by the inverse of the relative error *R*. When performing MLE, all the distributions of the parameters were assumed; Gaussian and the standard deviation were automatically inferred, while, when performing MCMC, we always checked the shape of the distribution to be a Gaussian, especially when performing the linear regressions 6 and 7 in the indirect models. Note that an increase in precision and accuracy corresponds to a decrease in the percentage given; in other words, high accuracy and precision correspond with low values of *R* and *P*.

**Figure 3:**
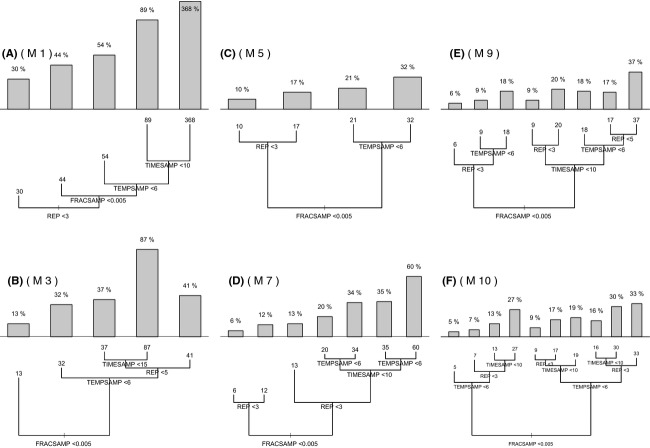
The results of the classification and regression tree (CART) analysis (Ripley 2007) of the relative error of the estimates of activation energy. The number at the leaves of the tree indicates the mean percentage value of the relative error of the estimate (see expression 8) over all the simulated experiments, following partitioning of the data in the manor specified by the tree. The threshold above each node indicates the split criterion used to separate the data. To each tree is associate a bar chart showing the mean percentage value of each leaf. The six panels correspond to six of the models specified in Table 1: model M1 (panel A), M3 (panel B), M5 (panel C), M7 (panel D), M9 (panel E), and M10 (panel F).

We then applied classification and regression tree analysis (CART) (Ripley 2007) to the absolute value of the relative error of the estimates of activation energy (the response variable) for each of the methods in Table 1, in order to assess the relative importance of different experimental factors (the explanatory variables) and their interaction (see Fig. 3). A regression tree is constructed by repeated splits of the data into mutually exclusive groups. Each split is defined by values less than some chosen value of one of the experimental factors. At each split, the data are partitioned into two groups as homogenous as possible. Each group is distinguished by the mean of the absolute value of the relative error of the estimate of activation energy and the values of the experimental factors that define it (De'ath and Fabricius 2000; Ripley 2007). Splits are chosen in order to minimize the sum of squared error between the observation and the mean in each node of the tree. The splitting procedure is then applied to each group separately partitioning the response into homogeneous groups and keeping the tree sensibly small. Appropriate tree size is determined setting a threshold in the reduction in homogeneity measure (De'ath and Fabricius 2000). Regression trees are a powerful tool for their capacity of interactive exploration and description of different subsets of the data and are often used instead of more classic linear model analysis (De'ath and Fabricius 2000).

### Case study

As a case study, we present data from a microcosm experiment (Leary and Petchey 2009) in which time series of abundance were collected along a gradient of six different temperatures between 18 and 28°C, where there were three replicates and TEMPSAMP = 6 (please see Leary and Petchey 2009 for supplementary detail). In this case study, the fraction of habitat searched (FRACSAMP) and the frequency of sampling (TIMESAMP) were variable, the latter depending on the temperature and the former depending on the observed density; this was accounted for in the likelihood functions. We estimated the activation energy of the protist species *Paramecium caudatum* in these microcosm experiments using methods M1, M2, M7, M8, and M10 (see Table 1 for definitions). Methods M7, M8, and M10 were used because we found them to be the most effective in estimating activation energy. Methods M1 and M2 (using the phenomenological likelihood 15, section “Likelihoods and inference”) in Supporting information were included to act as a comparison with the best performing methods because we wanted to investigate how important their lack of accuracy and precision could be when estimating activation energy (see Fig. S2). We also found that real data do not strictly obey to the theory presented in (Savage et al. 2004) for carrying capacity (see Fig. 6B); for this reason, while using model M10, we implemented a likelihood with two different activation energies, one for growth rate (*E*_*A,r*_) and one for carrying capacity (*E*_*A,K*_).

## Results

Activation energy was estimated with a wide range of accuracies across the different experimental conditions and inference methods considered, varying from high accuracy (relative error estimates being within <5% of the mean value on average) to low accuracy (relative error estimates being >300% of the average; Fig. 3). The fraction of the habitat sampled, FRACSAMP, was the most important experimental factor influencing the accuracy of activation energy estimates, as revealed by FRACSAMP consistently being the first split in five of six CART analysis (Fig. 3). An exception was when using method M1 (Fig. 3A), the phenomenological likelihood (equation 15, section “Likelihoods and inference”) in Supporting information for parameter inference, which in general, produced relatively inaccurate estimates of activation energy. Therefore, for most methods, sampling >0.5% of the habitat leads to the biggest improvement in accuracy (decrease in relative error *R*) in the estimation of activation energy across all experimental factors. Also for the indirect methods which use carrying capacity as a parameter to infer activation energy (methods M2, M4, M6, and M8 in Table 1) the fraction of habitat searched is the most important experimental factor influencing the accuracy of activation energy estimates (see Fig. S1).

After FRACSAMP, there was no consistent ordering in the rank importance of the other experimental conditions across the different inference methods (Fig. 3). The number of different temperatures used along a temperature gradient and the number of replicates per experiment were both used for the second split in the classification trees, depending on the inference method used. For the number of replicates, accuracy was significantly lower for experiments with only one replicate than for those with more than one replicate. For example, when the fraction of habitat searched is >0.005, having at least three replicates instead of only one increases the accuracy of the estimates of activation energy from 16% to 10% error for method M5, from 12% to 6% error for method M7, and from 13% to 6% error for method M9 (Fig. 3A–D, respectively). For the number of temperatures, accuracy was significantly lower when just three temperatures were used than when more than three temperatures were used. The number of times in the 15 days period that samples were taken (TIMESAMP) appeared to have the smallest effect, although we expect this was because even the least frequent sampling still included low, medium, and high population densities in the time series. Replication also interacts with other factors such as the size of the temperature gradient (TEMPSAMP) to influence the accuracy of the estimates. For example, at low FRACSAMP, increasing the number of temperatures at which experiments are conducted will not increase the accuracy of estimates of activation energy when only one replicate is used per temperature when using indirect methods (Fig. 3D). However, having more temperatures will improve the estimate of activation energy when using a direct method (Fig. 3F).

**Figure 4:**
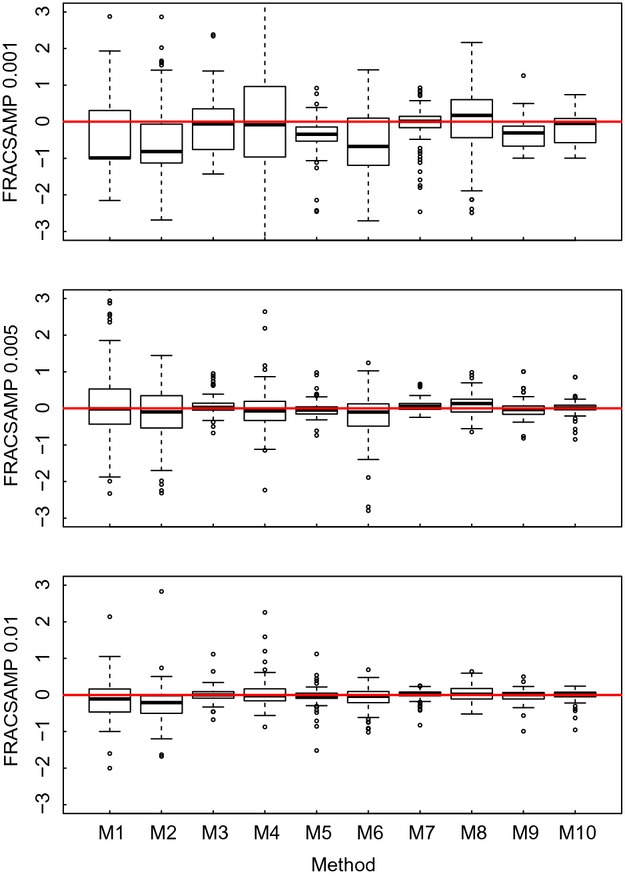
The variation in the relative error of each model indicated in Table 1 for different FRACSAMP, for experiments with one replicate, for each activation energies used in simulated data (*E*_*A*_ = 0.2–1.2 eV) for all values of TIMESAMP (5,10,15) and TEMPSAMP (3,6,11). The y axis displays percentage values of relative error. The black lines indicate the medians, and the boxes demarcate the 25–75% intervals. The whiskers extend up to one and a half times the interquartile range. The red line shows the maximum precision (i.e., estimated value = true value).

Taking into account, the observation error in the inference method increased the accuracy of estimates of activation energy when inferring it indirectly for carrying capacity (mean relative error of method M6 of 45% vs. mean relative error of M8 is 36%) and growth rate (mean relative error of method M5 is 16% vs. mean relative error of M7 is 11%). However, it led to only a minor improvement when inferring activation energy directly (mean relative error of method M9 is 10.6% vs. mean relative error of M10 is 10.3%). Estimates of activation energy are generally more accurate when estimated using MCMC parameter inference than using MLE, although sampling a larger fraction of the microcosm can clearly be used to compensate for this (see Fig. 4). Among the indirect MCMC methods, more accurate estimates of activation energy were obtained using the inferred growth rate rather than carrying capacity, and accounting for observational error improved these estimates further. These improvements were made with the inevitable cost of computational time (Table 1).

**Figure 5:**
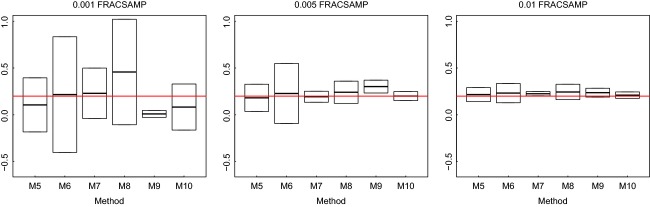
Example of different estimates of activation energy (see expression 8) for all the methods based on MCMC (as indicated in Table 1) for simulated data (TEMPSAMP = 6, TIMESAMP = 5 REP = 3). The black lines indicate the mean of the estimate, and the boxes demarcate the 95% confidence intervals of 1000 samples taken from the Markov chain. The red line shows the real parameter used for simulations (*E*_*A*_ = 0.2 eV).

Figure 3 shows the absolute value of the relative error of the estimates of activation energy; however, this does not indicate the degree to which the methods are over or underestimating activation energy. This is conveyed in Figs. 5. These results imply that for most of our methods, the true activation energy lies toward the center of the predicted probability distribution for that parameter. An exception is direct inference method M9 in which appears to consistently underpredict activation energy at low sampling intensities, which appears to be corrected by taking into account sampling error in method M10. Given the inferior performance of the MLE methods and the dominance of FRACSAMP, we only describe how the precision of estimates is affected by FRACSAMP for the MCMC methods. The most precise estimates of activation energy tend to be obtained using either the direct methods or the indirect methods on growth rate only with sampling error correction (Fig. 5 M7, M9, M10; the results illustrated in this figure are representative of what we observed for other sets of experimental conditions). In general, the most precise estimates were obtained using the direct methods (M9 and M10) which combine information on both growth rates and carrying capacities. Implementing the sampling error correction also tends to increase the precision of the estimated activation energies (Fig. 5). Interestingly, direct methods (M9 and M10) are clearly more sensitive to changes in the experimental conditions, as shown by the largest number of statistically significant branches in the regression trees (Fig. 3E and F).

**Figure 6:**
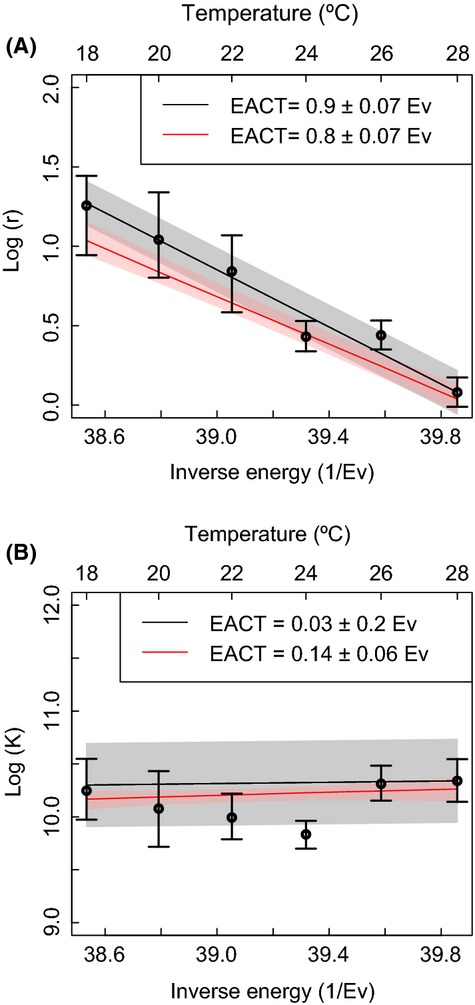
Estimates of the logarithm of the growth rate (panel A) and carrying capacity (panel B) of *Paramecium caudatum*. The error bars show the 95% confidence interval of the estimates obtained at each temperature separately. The black continuous line and shaded area represent the estimate of activation energy and the 95% confidence interval of the estimate of activation energy obtained from a weighted linear regression from the values observed at each temperatures (methods M7 for panel A and M8 for panel B, for the methods, see Table 1). The red line and shaded area are the mean and 95% confidence interval of the estimate obtained with method M10 (as in Table 1) with two different activation energies.

When used on real time series data, inferred population growth rate is linearly related to the inverse of temperature, with a negative slope given by the activation energy, as predicted by metabolic theory (Savage et al. 2004; Fig. 6A). In contrast, the temperature dependence of carrying capacity does not follow the theory (which predicts a positive relationship, Savage et al. 2004), showing no clear directional relationship with temperature (Fig. 6B). For the best performing methods in our simulation experiments (methods M7, M8, and M10), the direct and indirect methods produce different estimates of activation energy. The estimate for population growth rate from the direct method is slightly lower (*E*_*A*_ = 0.8 eV) than the estimate obtained indirectly (*E*_*A*_ = 0.9 eV). For the temperature range we considered, this difference leads to the largest contrast between predicted growth rates at *T* = 28°C, where the difference is roughly 1 day^−1^. Differences in the the mean estimates of activation energy of carrying capacity using direct and indirect methods do not lead to different predicted mean carrying capacities at different temperatures (largely because the estimated activation energy is close to zero). However, the precision of those predictions do contrast; for example, at *T* = 28°C, the standard deviation of the predicted carrying capacity is approximately 1000 individuals when using the direct method and is approximately 4500 individuals when using the indirect methods. An example of the different estimates obtained with direct and indirect methods at a given temperature (*T* = 22°C) is shown in Figure 2D. The activation energy of growth rate measured with the direct method is smaller than the one obtained with indirect methods and has a smaller error.

Applying the phenomenological methods leads to notable differences in the accuracy of the estimates of activation energy for the microcosm experiments. Using indirect method M1 (phenomenological) to estimate activation energy leads to an estimate, that is, 0.2 eV lower than that generated by indirect method M7 (0.7 eV compared to 0.9 eV, respectively; Fig. S2A). This difference translates to a difference in predicted growth rate at *T* = 28°C of 1.2 day^−1^. A similar difference is observed when estimating the activation energy of carrying capacity: indirect method M2 (phenomenological) gives an estimate, that is, 0.2 eV higher than that generated by indirect method M8 (0.03 eV compared to 0.2 eV, respectively; Fig. S2B). In this example, this could lead to a qualitatively different conclusion about whether carrying capacity is related to temperature, with the phenomenological method implying a positive relationship, whereas method M8 implies no relationship.

## Discussion

Our results revealed how experimental factors and parameter inference methods interact to influence the accuracy with which activation energy can be inferred. We found that the fraction of habitat searched is the most important factor in determining the accuracy of the estimates of activation energy. We also provided a list of inference methods from the least to the most accurate, for a set of experimental designs (see Fig. 4), including a classic phenomenological likelihood (Pascual and Kareiva 1996) where no information about demographic stochasticity was included, likelihoods that accounted for demographic stochasticity (Ross et al. 2006), and likelihoods that accounted for demographic stochasticity and sampling error (Ross et al. 2009). Inference methods that included the different sources of stochasticity improved the precision and the accuracy of the estimates of activation energy of at least one order of magnitude, for a given experimental design, especially when the fraction of habitat searched was small. The largest improvement in the accuracy of the estimates was obtained using a diffusion approximation (Ross et al. 2006, 2009) for continuous time stochastic processes. The use of such approximation enabled us to disentangle different sources of noise (demographic and sampling) and could be extended to more complex models. Another key improvement to the inference was fitting (directly) to all available data simultaneously. Moreover, taking into account the sampling error correction in direct methods, where the information of both temperature dependencies of growth rate and carrying capacity is taken into account, slightly improved the estimate of activation energy. Application of these simulation-based findings to real data suggests that although this direct method is more accurate, prior use of the indirect method is useful to reveal the functional form of the temperature dependency.

Comparison of the indirect and direct methods of inference revealed the unique strengths of each approach. Indirect methods are useful to identify the strengths and weaknesses of the different models describing single temperature time series. Once a suitable functional form is implemented, the temperature dependence of ecological parameters can be better inferred using direct methods; yet direct methods could be misleading if applied without having a clear understanding of the outcome of the indirect methods. For example, in our study, we based our simulations on a specific exponential function (Arrhenius law) scaled with a single parameter (activation energy). Different functional forms (such as hump-shaped functions) would have required a different implementation into direct methods. Similar approaches have been used in other modeling frameworks (Grimm et al. 2005; Smith et al. 2013) where parameter borrowing between different experiments is used to inform the global parameterization of the model (McInerny and Purves 2011; Sibly et al. 2013; Smith et al. 2013). The direct approach could be further generalized in more complex models such as food web models (Petchey et al. 2010) or stage-structured models (Ananthasubramaniam et al. 2011). When assessing the performance of different models against data, direct and indirect methods should be combined.

When using direct methods on time series data for *Paramecium caudatum*, we found that the estimates of growth rate at each temperature were affected by the estimates of carrying capacity, thus giving “neighborly advice” (McInerny and Purves 2011) on the temperature dependence of growth rate. The difference in estimation between direct and indirect methods led to large differences in predicted population dynamics (Fig. 2D). The thermal performance curves of *Paramecium caudatum* have been assessed only using indirect methods (using growth rate as reference parameter; Krenek et al. 2011), and several models have been proposed to capture the temperature dependence of microbial growth (Huang et al. 2011; Krenek et al. 2011). We provide a framework to test further the thermal performance of microbial organisms, combining the information of carrying capacity with the information on growth rate. Our methods could be used to compare different thermal performance curves in microbial experiments (Angiletta 2006) and be further tested with different processes such as feeding rates (Rall et al. 2009; Englund et al. 2011; Fussmann et al. 2014) and with different environmental variables such as nutrient concentration (Weisse et al. 2002).

The use of stochastic models such as continuous birth and death processes (McKane and Newman 2004; Black and McKane 2012) provides a probabilistic framework to derive inference schemes from (Ross et al. 2006, 2009) and provides insight into the determinants of population dynamics (Black and McKane 2012). Despite the lack of a mathematical expression for the probability distribution of the populations in our study, the use of approximations, such as the diffusion one, provided an analytical expression for the first two moments of the population probability distribution (Ross et al. 2009; Ross 2012). Extending stochastic models to different systems with more than one species is analytically daunting, but numerically feasible. The mechanistic understanding of more complex multispecies models is then limited by their mathematical intractability. When it is not possible to obtain analytical expressions for population probability distributions, the Bayesian framework can be still used with numerical techniques such as particle filters (Ionides 2003; Ionides et al. 2006) or approximate Bayesian computation (Beaumont 2010). Those methods simulate directly, with a given precision, the likelihood of the model at each iteration of the Markov chain (Hartig et al. 2011). Markov chain Monte Carlo methods are more computationally demanding than classic maximum likelihood estimation, especially when implementing state space models; however, they give a more complete estimation of the probability distribution of the parameters of the model and of their correlation, especially when the distribution of those parameters is not Gaussian.

We chose not to vary *N* for simplicity in this study although we expect that changes in *N* to influence our estimates of activation energy in two ways. Firstly, varying *N* by large amounts (e.g., over an order of magnitude) will significantly change the time the populations take to approach equilibrium, meaning that an adjustment to the sampling design (frequency and intensity) may be needed to obtain a good characterization of the population dynamics. Secondly, the difference between *N* and *K* determines the magnitude of demographic fluctuations in the population (see section “Details on the formulation of the stochastic model”) in Supporting information. As a consequence, we expect that differences in *N* would lead to differences in demographic noise which could influence the precision with which we can estimate activation energy. However, the temperature dependence of growth rate and carrying capacity is not dependent on *N* in our simulation experiments, and so we expect that, given an adequate amount of sampling and a sufficiently large temperature range, our conclusions about the effects of likelihood methods and experimental design on estimates of activation energy will be insensitive to our choice of *N*. Again for simplicity, we assumed that density dependence only influences the probability of births while in reality, it commonly influences the probability of both. In section “Details on the formulation of the stochastic model”, in Supporting information we give the formulations for the more general birth and death processes in which both birth and death rates depend on *N*. When combined, these lead to more free parameters, but identical formulations for the temperature dependence of population growth rate and carrying capacity; thus, our results would be unaffected.

Our methods could improve the development of the ecological theory aimed at understanding the temperature dependence of population rates (Brown et al. 2004; Amarasekare and Savage 2012) or inform debates about the precise value of activation energy (Glazier 2006). The use of classic indirect methods can be used as a first step in identifying reasonable functional forms for the temperature dependence of population parameters, as biologists have extensively performed for a variety of taxa (Gillooly et al. 2001; Savage et al. 2004; Amarasekare and Sifuentes 2012). Different models associated with different functional forms of the rate temperature relations have now been proposed (Brown et al. 2004; Knies and Kingsolver 2010; Amarasekare and Savage 2012), and those models, arising from the combination of data and theory, can be further tested using the direct estimation methods we describe here.

One of the remaining conundrums in population and community ecology is about predictive ability. Studies have shown that uncertainty in parameter estimates can preclude predictions of even the direction (increase or decrease) of the effects of a perturbation (Yodzis 1988; Wells et al. 2014) but also that more accurate estimates will provide better predictions (Novak et al. 2011). Our findings support the idea that considerable potential for improved predictive ability lies in improving inference methods, including using quite complex mathematics and fitting algorithms, as well as continuing to use appropriate experimental designs and sampling schemes. The resulting increases in accuracy are likely to be very important, given the documented high sensitivity of model predictions to variation in parameter values.

## Supporting Information

**Data S1.** Time series of the species Paramecium caudatum at different temperatures.

**Figure S1.** The results of the classification and regression tree (CART) analysis (Ripley 2007) of the relative error of the estimates of activation energy. The number at the leaves of the tree indicates the mean percentage value of the relative error of the estimate (see expression 8) over all the simulated experiments, following partitioning of the data in the manor specified by the tree. The threshold above each node indicates the split criterion used to separate the data. To each tree is associate a bar chart showing the mean percentage value of each leaf. The four panels correspond to four of the models specified in Table 1: model M2 (panel A), M4 (panel B), M6 (panel C), and M8 (panel D).

**Figure S2.** Estimates of the logarithm of the growth rate (panel A) and carrying capacity (panel B) of *Paramecium caudatum*. The error bars show the 95% confidence interval of the estimates obtained at each temperature separately using the phenomenological likelihood 15. The red continuous line and shaded area represent the estimate of activation energy and the 95% confidence interval of the estimate of activation energy obtained from a weighted linear regression from the values observed at each temperatures (methods M1 for panel A and M2 for panel B, for the methods, see Table 1). The black line and shaded area represent the estimate of activation energy and the 95% confidence interval of the estimate of activation energy obtained from a weighted linear regression from the values observed at each temperatures obtained using likelihood 18 (methods M7 for panel A and M8 for panel B, for the methods, see Table 1) as shown in Figure 6.
