# Supplementary material for: Inferring the temperature dependence of population parameters: the effects of experimental design and inference algorithm
Source: Ecol Evol. 2014 Dec 2;4(24):4736–50. doi: 10.1002/ece3.1309 (PMC4278823; doi:10.1002/ece3.1309)
Supplement: Supplementary file 1 — Data S1. Time series of the species Paramecium caudatum at different temperatures. [file ece30004-4736-sd1.docx]

**5 Supplementary Information**

**5.1 Details on the formulation of the stochastic model**

The most general Verhulst-like stochastic birth and death process (Nåsell 2001) is given by Population birth and death rates $B(n)$ and $D(n)$ defined by

$$B\left( n,\theta\right)=\theta_{1}n\left( 1-\frac{\theta_{2}n}{N} \right),$$

$D\left( n,\theta\right)=\theta_{3}n\left( 1+\frac{\theta_{4}n}{N} \right).$ (9)

Where $n$ is the population abundance and can take values in the set$0,1,\ldots,N$. The parameters $\theta_{1}$ and $\theta_{3}$ are the per capita intrinsic birth and death rates. The parameters $\theta_{2}$and$\theta_{4}$are a measure of the effects of intraspecific competition on birth and death rate respectively and are dimensionless. All the parameters of the model ($\boldsymbol{\theta}$) follow the Arrhenius law (equation 2). Process 9 is more general than process 1 as it includes density dependence also in the death rate. The carrying capacity of process 9 is given by $K=\frac{\theta_{1} -\theta_{3}}{\theta_{1}\theta_{2} + \theta_{3}\theta_{4}}N$. $N$ is the population density at which there is zero probability of births (see section 2) and $N-K$ represent the maximum size of fluctuations associated with demographic stochasticity (Nasell 2001). In the more general process it is mathematically convenient to scale the strength of density dependence for both birth and death rates with the same parameter, $N$: the population size at which the probability of births is zero. This makes it more mathematically tractable to calculate the expectation of the population size and the diffusion approximation for population density. In reality we expect that birth and death probabilities would scale differently with population size, but this could be accommodated by differences in the parameters $\theta_{2}$ and $\theta_{4}$ in process 9

The associated stochastic differential equation (SDE) of the process 9 is

$\frac{dn\left( t \right)}{dt}=F\left( n\left( t \right);\theta\right)+\sqrt{H\left( n\left( t \right);\theta\right)}\frac{dW\left( t \right)}{dt},$ (10)

where, from 9, we defined two associated functions $F\left( n;\theta\right)=B\left( n;\theta\right)-D(n;\theta)$ and $H\left( n;\theta\right)=B\left( n;\theta\right)+D(n;\theta)$, and where $W$ is the standard Wiener process, where $\Delta W\left( t \right)=W\left( t+\Delta t \right)-W(t)$ has a normal distribution with 0 mean and variance given by $\Delta t$ (Allen & Allen 2003; Gardinier 2009). The deterministic term in the SDE 10 is the classic logistic equation (see equation 3 in the main text). It is important to stress that for equations 10 and 3 $n(t)$ takes continuous values in the interval $[0,N]$. The stochastic term in equation 10 is due to random variations in the birth and death rates (demographic stochasticity).

Another way of looking at process 9 is to describe it (Gardinier 2009) by the following master equation

$\frac{dP\left( n,t \right)}{dt}=D\left( n+1;\theta\right)P\left( n+1,;\theta\right)+D\left( n-1;\theta\right)P\left( n-1,;\theta\right)-H(n;\theta)P(n;\theta)$, (11)

where $P(n,t)$ is defined as the probability of having $n$ individuals at time $t$ and $H\left( n;\theta\right)=B\left( n;\theta\right)+D(n;\theta)$. In analogy with chemical kinetics, we call the functions $B$ and $D$ the reaction hazards and $H$ the cumulative hazard of the process (Wilkinson 2006). A detailed mathematical analysis of equation 11 is usually intractable, but is straightforward to simulate the time evolution of the system given the rates 9. The most common discrete event simulation procedure is known as the *Gillespie algorithm* (Gillespie 1976; Gillespie 1977).

We obtain process 1 from the more general process 9 by putting $\theta_{4}=0$ i.e., assuming that intraspecific competition affects only births. Conveniently for our study here, Ross, Pagendam & Pollett 2009 derived an approximation for the BDP 1 which gives the probability of observing a particular number of individuals as a Gaussian distribution with time dependent mean and variance (assuming the maximum population size is sufficiently large $>1000$; Ross, Pagendam & Pollett 2009))

$P_{g}\left( X=x;t,\theta\right)=\frac{1}{\sqrt{2\pi\sigma\left( t;\theta\right)^{2}}}\exp\left( -\frac{x-n\left( t;\theta\right)}{2\sigma\left( t;\theta\right)^{2}} \right)$ (12)

where the mean of this distribution ($n(t;\theta)$) is given by the solution of the logistic equation 3

$n\left( t,n_{0};\theta\left( T \right) \right)=\frac{K\left( T \right)n_{0}e^{r\left( T \right)t}}{K\left( T \right)+n_{0}(e^{r\left( T \right)t}-1)}$ (13)

and the variance is given by

$\sigma\left( t,n_{0};\theta\left( T \right) \right)=NM_{t}^{2}\int_{0}^{t} H(n(s,n_{0},\theta\left( T \right))/N;\theta)M_{s}^{-2}ds$ (14)

where $M_{s}=\exp\int_{0}^{s} B_{s}ds$ and ${B_{s}=F}^{'}(n\left( s \right)/N)$, (Ross, Taimre & Pollett 2006; Ross, Pagendam & Pollett 2009). In section 5.2 we describe how we used this approximation to compute the probability of the population being in a particular state given the model parameters.

**5.2 Likelihoods and inference**

Here we describe the likelihood functions used for the inference of activation energy from population time series collected at different temperatures. For each temperature the data is given by$\boldsymbol{y}_{\boldsymbol{T}_{\boldsymbol{k}}}=(n_{0,k},t_{0};n_{1,k},t_{1};\ldots;n_{d,k},t_{d})$, where $d$ is the sampling effort (d=TIMESAMP) and $n_{i,k}$ is the number of individuals counted at time $t_{i}$ and at temperature$T_{K}$. A likelihood function whose arguments are the data and the parameters of process 1 is associated to each method described in table 1.

The first likelihood function we consider is classically used by ecologists in fitting models to time series data (Pascual & Kareiva 1996; Hilborn 1997)

$L_{phen}\left( \boldsymbol{y}_{\boldsymbol{T}_{\boldsymbol{k}}} | \boldsymbol{\Theta}\left( T_{k} \right) \right)=\sum_{i=1}^{d} \log\left[ P_{g}\left( X={\Delta n}_{i,k},\boldsymbol{\Theta}\left( T_{k} \right) \right) \right]$, (15)

where $\Delta n_{i,k}\left( t_{i} \right)=\log\left( n_{i,k} \right)-log(n(t_{i}-t_{i-1},n_{i-1,k};r\left( T_{k} \right),K\left( T_{k} \right))$ is the difference between the logarithms of the observed population densities and the predicted mean densities (see solution 13) at every time step, and where the parameters estimated, for every temperature, are $\boldsymbol{\Theta}\left( T_{k} \right)=(r\left( T_{k} \right), K\left( T_{k} \right),\sigma_{k})$ i.e., the growth rate and the carrying capacity together with a variance $\sigma_{k}$. We refer to this as the phenomenological likelihood function because it assigns high likelihood to parameters that capture the phenomenon of logistic population growth without accounting for the effects of the parameters on the demographic stochasticity observed in the population, or for sampling error.

All other likelihood functions incorporate the mathematical derivation of Ross, Pagendam & Pollett 2009 for the probability of a population having a particular size at a particular time when following the stochastic birth death process 1. If we do not account for sampling error and infer activation energy indirectly then the likelihood function incorporating the probability distribution 12 with variance 14 is

$L_{1}\left( \boldsymbol{y}_{\boldsymbol{T}_{\boldsymbol{k}}} | \theta'\left( T_{k} \right) \right)=\sum_{i=1}^{d} \log\left[ P_{g}\left( X=n_{i,k};t_{i},\theta\left( T_{k} \right) \right) \right]$, (16)

where$\theta_{1}^{'}(T_{k}) = log(N/\theta_{1}\left( T_{k} \right)\theta_{2}(T_{k})) = log(K(T_{k})/r(T_{k}))$, $\theta_{2}^{'}(T_{k})=log(\theta_{3}(T_{k})-\theta_{1}(T_{k}))=log(r(T_{k}))$, and $\theta_{3}^{'}(T_{k})=log(N/\theta_{2}(T_{k}))$. This parameterization is particularly convenient for the indirect estimation of activation energy because it naturally provides the logarithm of the parameters as in expressions 6 and 7. More importantly, these specific parameterizations improved the performance of the inference algorithms, in terms of the rate at which they converged on the correct answer, because they largely removed parameter correlations. We did this for all methods involving likelihoods 16, 18, 19 and 20.

One key decision to take while doing inference is whether to account for sampling error. Accounting for sampling error requires us to infer the actual population size at each sampling time given the number of individuals observed in each sample (Cappé, Moulines & Ryden 2005). As we modeled the sampling process using a Poisson distribution, we can include into likelihood function 16 a correction account of the form

$P_{f}\left( X = n, \Lambda\right)= f\Lambda^{fn}\frac{e^{-f\Lambda}}{fn!}$, (17)

describing the probability of observing a population of $n$ individuals when the actual size of the population is is $\Lambda$ and the fraction of habitat searched is $f$ (FRACSAMP = $f$). We can account for sampling error by adding a correction term of the form 17 to likelihood 16 which becomes

$L_{2}\left( \boldsymbol{y}_{\boldsymbol{T}_{\boldsymbol{k}}} | {\bar{\boldsymbol{y}}}_{\boldsymbol{T}_{\boldsymbol{k}}},\theta^{'}\left( T_{k} \right) \right)= \sum_{i=1}^{d} \log\left[ P_{g}\left( X=\bar{n}_{i,k};t_{i},\theta\left( T_{k} \right) \right) \right]+ log[P_{f}(X=n_{i,k},\bar{n}_{i,k})],$ (18)

where ${\bar{\boldsymbol{y}}}_{\boldsymbol{T}_{\boldsymbol{k}}}(\bar{n}_{0,k}, t_{0}; \bar{n}_{1,k}, t_{1};\ldots; \bar{n}_{d,k},t_{d})$ is a vector of latent variables giving the inferred expected population sizes $\bar{n}_{i,k}$ at time $t_{i}$ and temperature $T_{k}$.

It is straightforward to extend likelihood functions 16 and 18 to allow activation energy to be inferred directly by incorporating all time series at different temperatures i.e., $\boldsymbol{Y}_{\boldsymbol{T}}=\{\boldsymbol{y}_{\boldsymbol{T}_{\boldsymbol{0}}},\boldsymbol{y}_{\boldsymbol{T}_{\boldsymbol{1}}};\ldots;\boldsymbol{y}_{\boldsymbol{T}_{\boldsymbol{q}}}\boldsymbol{\}}$. We obtain a more direct method to estimate activation energy by summing likelihoods 16 and 18 over all possible temperatures of the gradient

$L_{1}^{D}(\boldsymbol{Y}_{\boldsymbol{T}}| \theta_{0}^{'},\theta_{4}) =\sum_{k=1}^{q} L_{1}\left( \boldsymbol{y}_{\boldsymbol{T}_{\boldsymbol{k}}} | \theta^{'}\left( T_{k} \right) \right)$ , (19)

$L_{2}^{D}\left( \boldsymbol{Y}_{\boldsymbol{T}} \right|{\bar{\boldsymbol{Y}}}_{\boldsymbol{T}}\boldsymbol{,}\theta_{0}^{'},\theta_{4}) =\sum_{k=1}^{q} L_{2}(\boldsymbol{y}_{\boldsymbol{T}_{\boldsymbol{k}}}|{\bar{\boldsymbol{y}}}_{\boldsymbol{T}_{\boldsymbol{k}}},\theta'(T_{k}))$ , (20)

where $q$is the size of the temperature gradient (q=TEMPSAMP) and ${\bar{\boldsymbol{Y}}}_{\boldsymbol{T}}\boldsymbol{=\{}{\bar{\boldsymbol{y}}}_{\boldsymbol{T}_{\boldsymbol{0}}}\boldsymbol{,}{\bar{\boldsymbol{y}}}_{\boldsymbol{T}_{\boldsymbol{1}}}\boldsymbol{,\ldots,}{\bar{\boldsymbol{y}}}_{\boldsymbol{T}_{\boldsymbol{q}}}\boldsymbol{\}}$ are all the latent variables i.e., the inferred expected population sizes at all temperatures. In this case we infer $\theta_{0}^{'}$ i.e., the same parameters of the indirect likelihoods at the reference temperature $T_{0}$, and a fourth parameter $\theta_{4}=\log\left( E_{A} \right)$ which provides directly the information about the activation energy of the model. Note that fitting the more general model described by process 9 would require different likelihood functions from the one used in models M3-M10. Specifically adding a density dependence on the death rate would produce a different expression for the variance of population density (equation 14).

We used two different computational algorithms to estimate the most likely model parameters, one that seeks the maximum likelihood estimate of the parameters (MLE) and one that infers the joint probability distribution of the parameters given the data.

- Details on the MLE optimization methods: MLE was performed using function mle2() from the package bbmle in R (Bolker 2013), using the search method simulated annealing (SANN) (Kirkpatrick, Gelatt & Vecchi 1983) to maximize the likelihood function in parameter space. MLE was performed using two likelihood functions only, 15 and 16, because attempts to employ MLE to use the other likelihood functions were computationally unfeasible.

Simulated annealing is a stochastic optimization technique which enables to find low cost configurations while still exploring the parameter space (Kirkpatrick, Gelatt & Vecchi 1983). We choose SANN because, among the available optimization methods of the function optim() used by the function mle2(), SANN allowed likelihood estimation to be made where other search algorithms failed to optimize. MLE requires start values from which the search algorithm can begin to search the parameter space. These fitted values where given as normally distributed around the known actual parameters values with variance 1% of the actual value.

- details of the MCMC and filzbach

We inferred the joint probability distributions of the parameters using Markov Chain Monte Carlo sampling with the Metropolis-Hastings algorithm (Chib & Greenberg 1995), implemented using the software Filzbach (Filzbach 2013). We used uniform uninformative priors for all the parameters using likelihoods 16, 18, 19 and 20. We did not use this approach with likelihood 15 for brevity, after observing the poor parameter estimates using that approach in preliminary analyses. Multiple chains of varying lengths were run initially to check for convergence on a single parameter probability distribution and on the rate of convergence before deciding on a burn in length of 5 million iterations and a sampling period of 5 million iterations. Chains were subsampled every 5000 iterations to remove autocorrelation before analyzing the parameter distributions. MCMC sampling is particularly attractive for parameter-rich problems. In fact the algorithm accepts any change in the parameters that increases the likelihood, but it also probabilistically takes changes that decrease the likelihood, according to the so-called “Metropolis criterion” (Chib & Greenberg 1995). This latter behavior is particularly important in nonlinear problems, because it allows the algorithm to escape from local maxima of the likelihood, and find the global maximum.

**5.3 Supplementary Results**

See Figures S1 and S2.
