# Supplementary figures and images for: Inferring the temperature dependence of population parameters: the effects of experimental design and inference algorithm
Source: Ecol Evol. 2014 Dec 2;4(24):4736–50. doi: 10.1002/ece3.1309 (PMC4278823; doi:10.1002/ece3.1309)

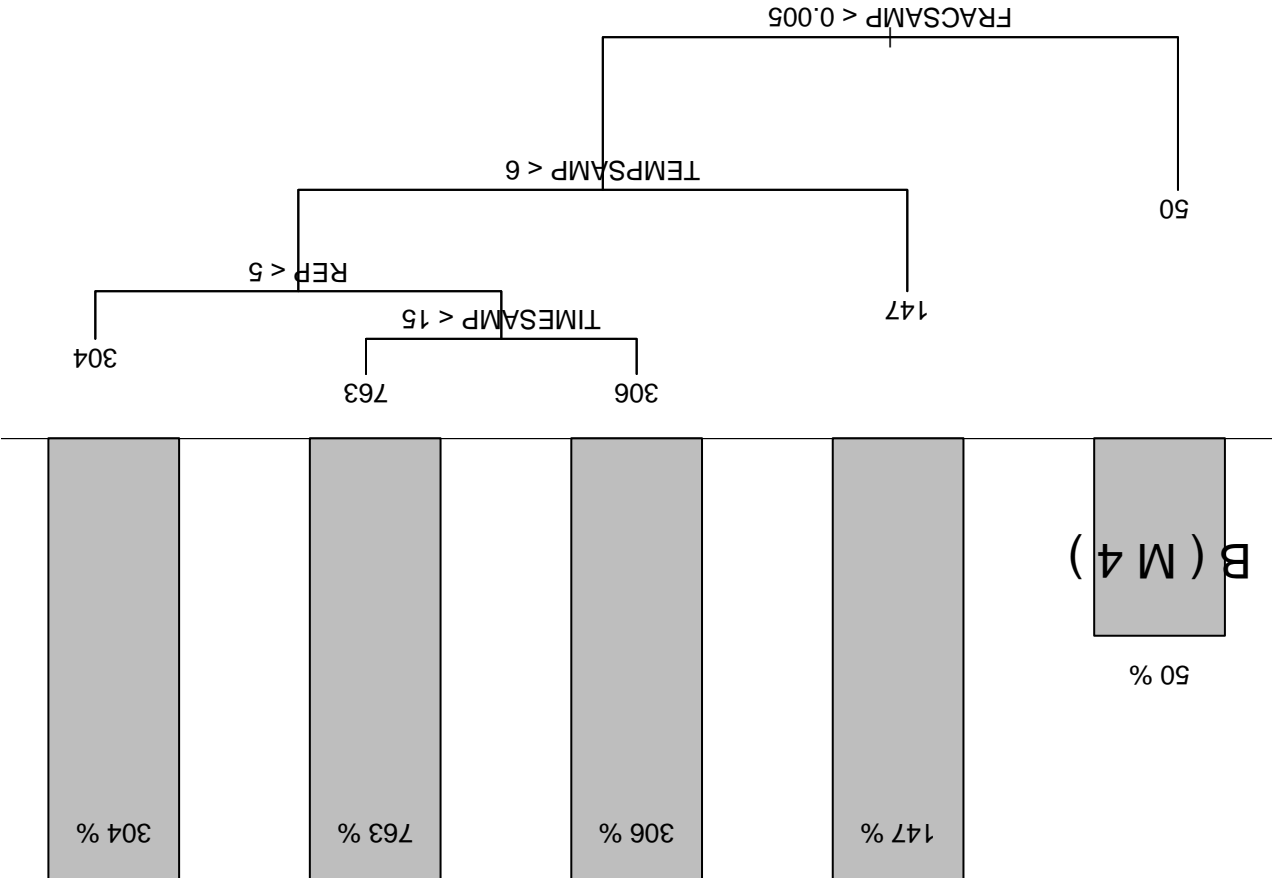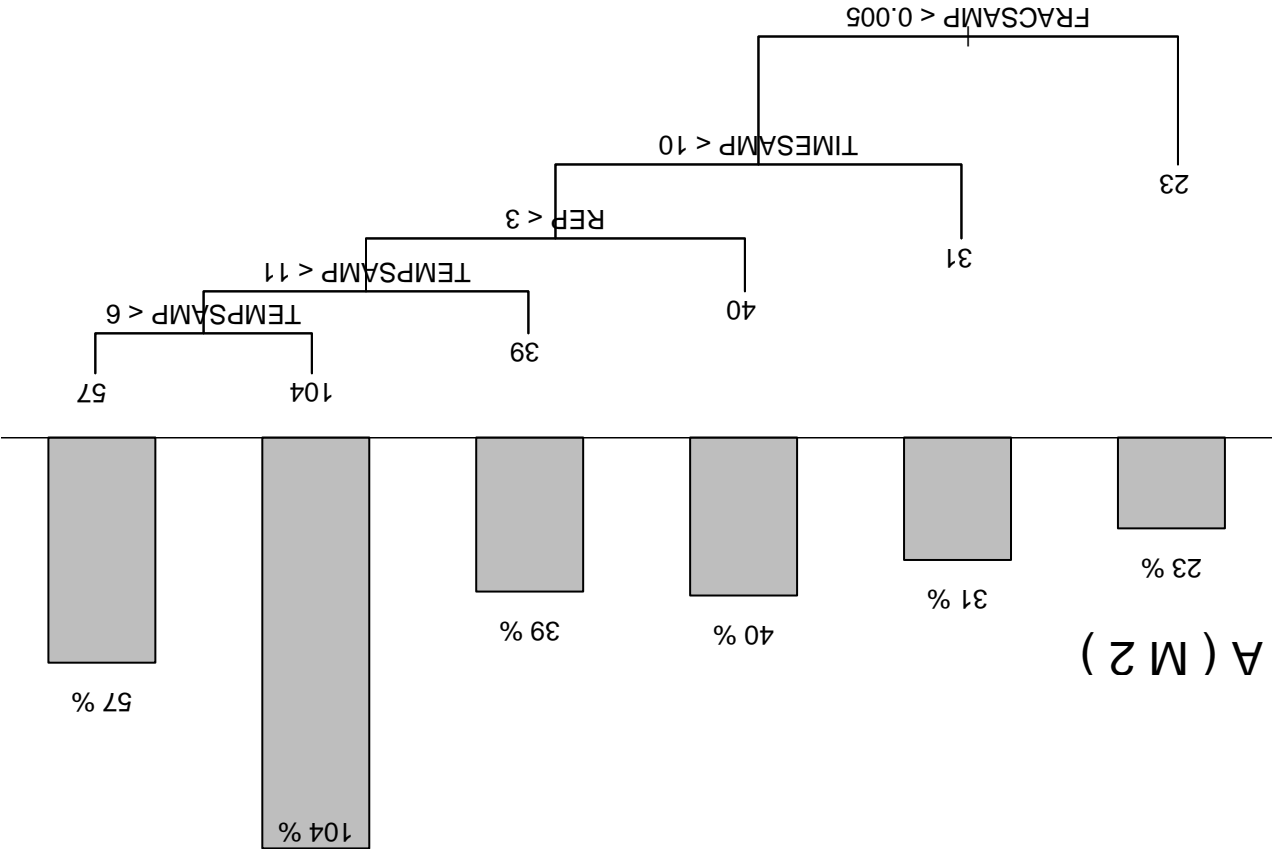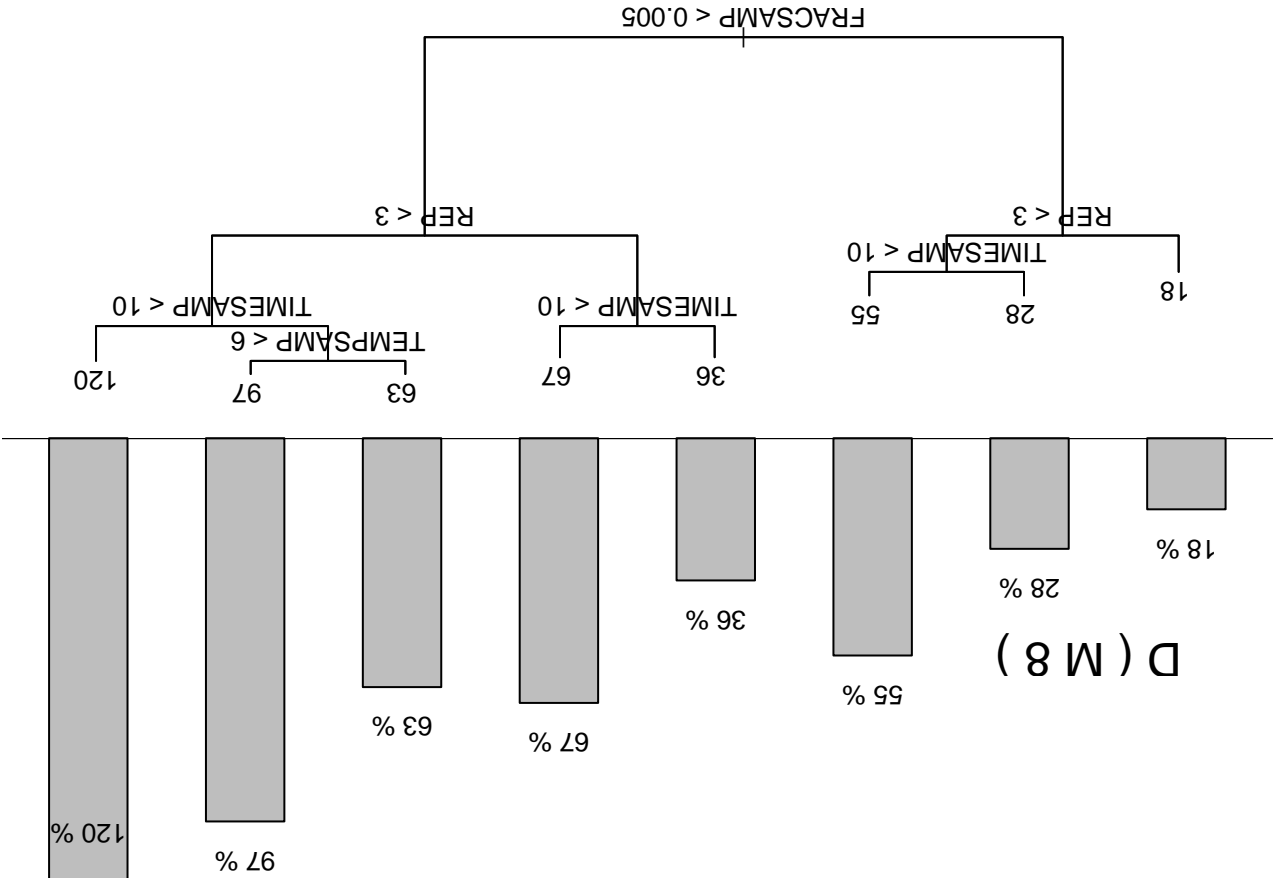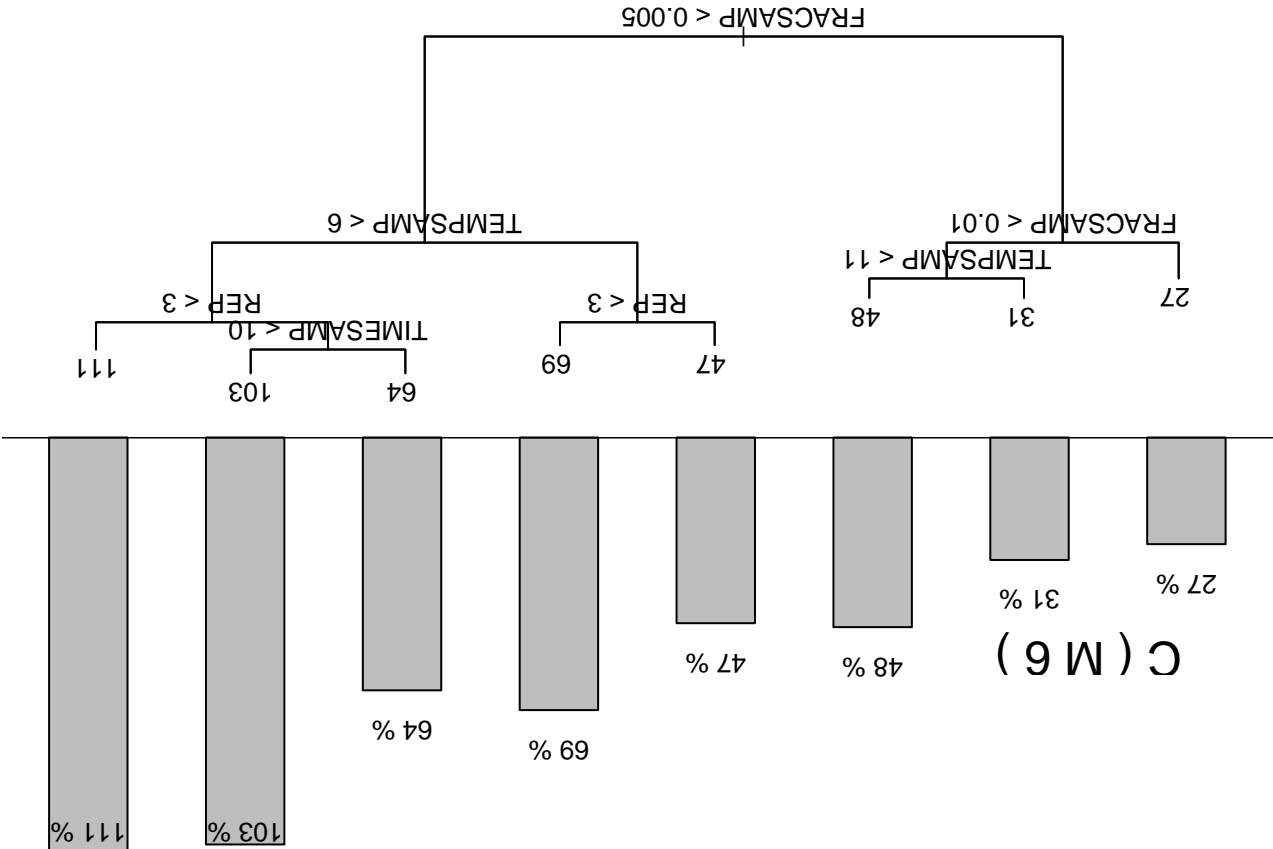

Supplement: Supplementary file 3 — Figure S1. The results of the classification and regression tree (CART) analysis (Ripley 2007) of the relative error of the estimates of activation energy. The number at the leaves of the tree indicates the mean percentage value of the relative error of the estimate (see expression 8) over all the simulated experiments, following partitioning of the data in the manor specified by the tree. The threshold above each node indicates the split criterion used to separate the data. To each tree is associate a bar chart showing the mean percentage value of each leaf. The four panels correspond to four of the models specified in Table 1: model M2 (panel A), M4 (panel B), M6 (panel C), and M8 (panel D). [file ece30004-4736-sd3.pdf]

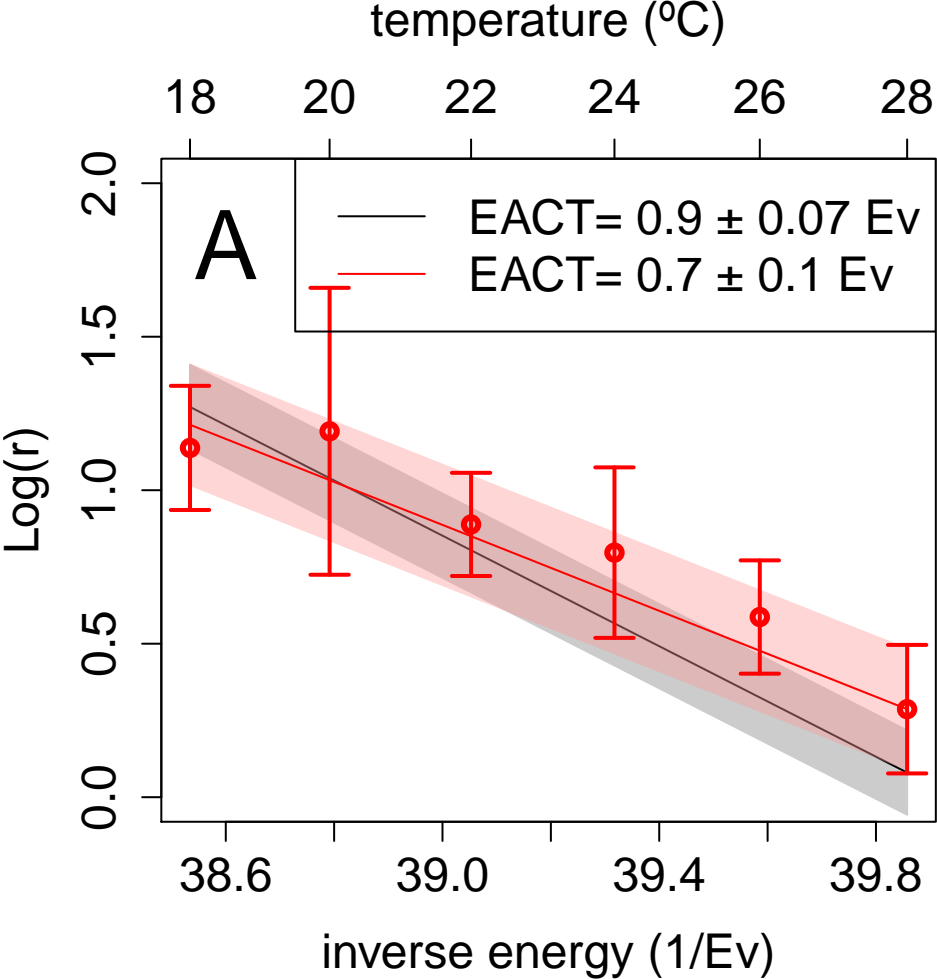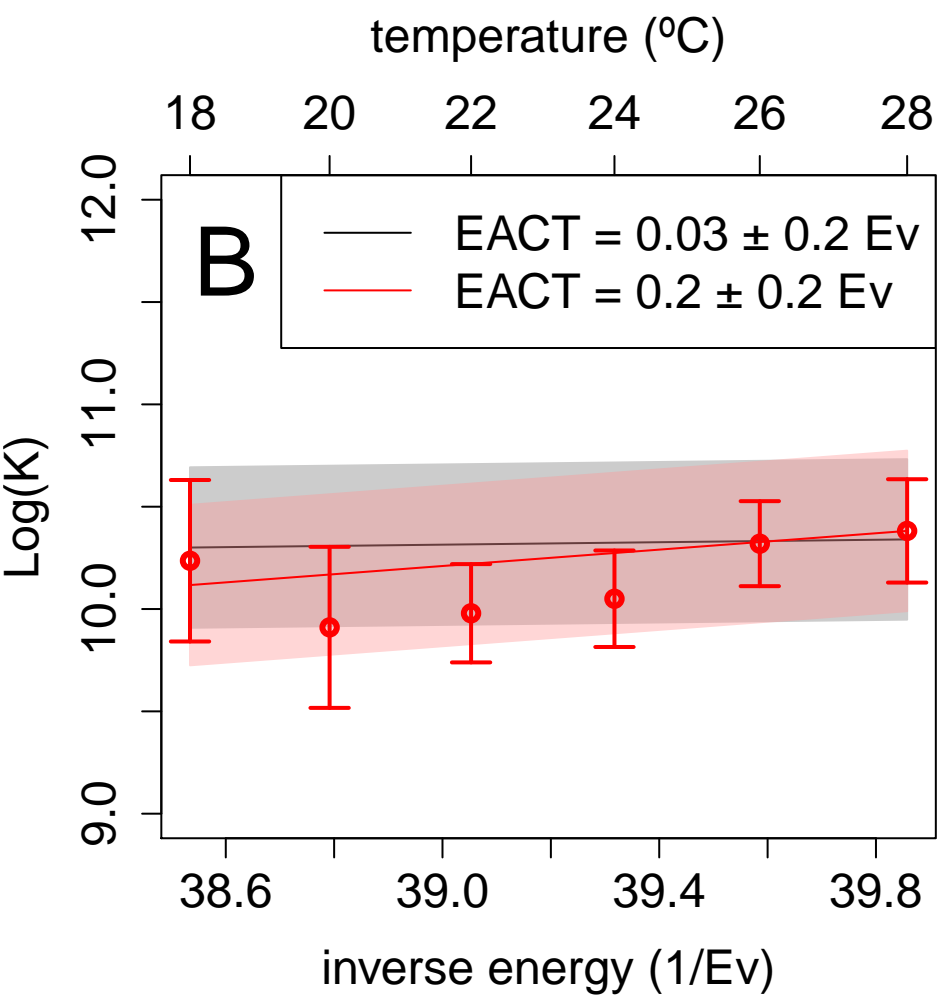

Supplement: Supplementary file 4 — Figure S2. Estimates of the logarithm of the growth rate (panel A) and carrying capacity (panel B) of Paramecium caudatum. The error bars show the 95% confidence interval of the estimates obtained at each temperature separately using the phenomenological likelihood 15. The red continuous line and shaded area represent the estimate of activation energy and the 95% confidence interval of the estimate of activation energy obtained from a weighted linear regression from the values observed at each temperatures (methods M1 for panel A and M2 for panel B, for the methods, see Table 1). The black line and shaded area represent the estimate of activation energy and the 95% confidence interval of the estimate of activation energy obtained from a weighted linear regression from the values observed at each temperatures obtained using likelihood 18 (methods M7 for panel A and M8 for panel B, for the methods, see Table 1) as shown in Figure 6. [file ece30004-4736-sd4.pdf]
